# Supplementary material for: Single cell transcriptional signatures of the human placenta in term and preterm parturition
Source: eLife. 2019 Dec 12;8:e52004. doi: 10.7554/eLife.52004 (PMC6949028; doi:10.7554/eLife.52004)
Supplement: Supplementary file 1. — Each row summarizes each 10X Genomics scRNA-seq library prepared and processed in this study: sample ID, number of cells detected after filtering, location of the tissue (BP = basal plate, PV = Placental Villi, CAM = chorioamniotic membranes), pregnancy condition (TNL = term no labor, TIL = term in labor, PTL = preterm labor), gender of the neonate, and total number of UMIs detected. [file elife-52004-supp1.docx]

| LibraryID | Total Cells | Location | Condition | Fetal Sex | Total UMI |
| --- | --- | --- | --- | --- | --- |
| s1DB | 2,574 | BP | TIL | Male | 15,122,851 |
| s1W | 2,984 | CAM | TIL | Male | 16,520,673 |
| s2DB | 2,313 | BP | TNL | Male | 7,897,722 |
| s2P | 2,490 | PV | TNL | Male | 13,393,737 |
| s2W | 2,905 | CAM | TNL | Male | 19,972,076 |
| s3DB | 2,340 | BP | TNL | Female | 9,158,823 |
| s3P | 2,546 | PV | TNL | Female | 21,869,861 |
| s3W | 2,038 | CAM | TNL | Female | 18,387,161 |
| s4DB | 3,165 | BP | TNL | Male | 19,265,820 |
| s4P | 3,007 | PV | TNL | Male | 24,326,111 |
| s4W | 2,691 | CAM | TNL | Male | 20,041,494 |
| s5DB | 4,629 | BP | TIL | Female | 16,519,752 |
| s5P | 3,012 | PV | TIL | Female | 14,238,071 |
| s5W | 2,011 | CAM | TIL | Female | 9,485,943 |
| s6DB | 3,723 | BP | PTL | Female | 25,713,932 |
| s6P | 1,238 | PV | PTL | Female | 12,637,713 |
| s6W | 4,826 | CAM | PTL | Female | 9,179,363 |
| s7DB | 2,945 | BP | PTL | Female | 24,058,875 |
| s7P | 2,711 | PV | PTL | Female | 27,913,872 |
| s7W | 3,221 | CAM | PTL | Female | 14,562,313 |
| s8DB | 3,054 | BP | PTL | Female | 17,932,348 |
| s8P | 4,246 | PV | PTL | Female | 46,082,098 |
| s8W | 5,232 | CAM | PTL | Female | 40,774,413 |
| s9DB | 3,992 | BP | TIL | Female | 20,688,976 |
| s9W | 4,013 | CAM | TIL | Female | 30,633,207 |
