# Supplementary material for: Single cell transcriptional signatures of the human placenta in term and preterm parturition
Source: eLife. 2019 Dec 12;8:e52004. doi: 10.7554/eLife.52004 (PMC6949028; doi:10.7554/eLife.52004)
Supplement: Supplementary file 6. — Data are given as medians with interquartile ranges (IQR) or as percentages (n/N). aOne sample missing data. [file elife-52004-supp6.docx]

|  | **TNL** | **TIL** | **PTL** |
| --- | --- | --- | --- |
| **Clinical parameters** | | | |
| Maternal age (years; median [IQR]) | 32 (28-35) | 25 (22-31.5) | 27 (23.5-29) |
| Body mass index (kg/m^2^; median [IQR]) | 31.2 (30.3-37.7) | 43.3 (36.9-44.1) | 32.5 (28.9-39.9) |
| Primiparity | 0% (0/3) | 66.7% (2/3) | 33.3% (1/3) |
| Cesarean section | 100% (3/3) | 66.7% (2/3) | 33.3% (1/3) |
| Gestational age at delivery (weeks; median [IQR]) | 39.6 (39.3-39.6) | 39.1 (38.8-39.9) | 35.1 (33.4-35.2) |
| Birthweight (g) | 4030 (3900-4160)^a^ | 3310 (3160-3405) | 2145 (1680-2220.5) |
| **Ethnicity** | | | |
| African-American | 66.7% (2/3) | 66.7% (2/3) | 100% (3/3) |
| Caucasian | 33.3% (1/3) | 0% (0/3) | 0% (0/3) |
| Other | 0% (0/3) | 33.3% (1/3) | 0% (0/3) |
