# Supplementary material for: Single cell transcriptional signatures of the human placenta in term and preterm parturition
Source: eLife. 2019 Dec 12;8:e52004. doi: 10.7554/eLife.52004 (PMC6949028; doi:10.7554/eLife.52004)
Supplement: Transparent reporting form [file elife-52004-transrepform.docx]

***eLife’s* transparent reporting form**

We encourage authors to provide detailed information *within their submission* to facilitate the interpretation and replication of experiments. Authors can upload supporting documentation to indicate the use of appropriate reporting guidelines for health-related research (see [EQUATOR Network](http://www.equator-network.org/%20)), life science research (see the [BioSharing Information Resource](https://biosharing.org/)), or the [ARRIVE guidelines](http://www.plosbiology.org/article/info:doi/10.1371/journal.pbio.1000412) for reporting work involving animal research. Where applicable, authors should refer to any relevant reporting standards documents in this form.

If you have any questions, please consult our Journal Policies and/or contact us: [editorial@elifesciences.org](mailto:editorial@elifesciences.org).

**Sample-size estimation**

- You should state whether an appropriate sample size was computed when the study was being designed
- You should state the statistical method of sample size computation and any required assumptions
- If no explicit power analysis was used, you should describe how you decided what sample (replicate) size (number) to use

Please outline where this information can be found within the submission (e.g., sections or figure legends), or explain why this information doesn’t apply to your submission:

At the inception of this study, no appropriate methods with realistic assumptions for scRNA-seq studies were available to perform power calculations to determine sample sizes. Most of the scRNA-seq studies are performed on very limited sample sizes and treat each single cell as an observation, which is usually adequate to determine the profile of a specific cell-type or differences between cell-types present in the same specimen (if >100 cells are observed for each cell-type, see Methods section).

To profile each study group (TNL term no labor, TIL term in labor, and PTL preterm labor), we chose a sample size of 3 independent specimens for each group (N=9). We reasoned that the pseudo-bulk aggregate for each cell-type can be modeled by a negative binomial distribution and that a similar approach to RNA-seq power calculations can be applied (see Methods section). This sample size also allowed us to estimate dispersion for each group with the negative binomial model implemented in DESeq2 that we use in our analyses. While not detailed in the manuscript, under these assumptions and using the “rnaseqsamplesizeweb” tool, we expected only to uncover changes in gene expression (FDR < 10%, power > 0.59 ) on those genes (m = 5,000, m1 =500) with a sufficiently high read count (lambda0 = 1000), a relatively low dispersion (phi0 = 0.2), and a high fold change (FC > 3). Note that we also have additional samples for each placental compartment, which helps to increase the power to detect differentially expressed genes across the study groups and the placental compartments.

**Replicates**

- You should report how often each experiment was performed
- You should include a definition of biological versus technical replication
- The data obtained should be provided and sufficient information should be provided to indicate the number of independent biological and/or technical replicates
- If you encountered any outliers, you should describe how these were handled
- Criteria for exclusion/inclusion of data should be clearly stated
- High-throughput sequence data should be uploaded before submission, with a private link for reviewers provided (these are available from both GEO and ArrayExpress)

Please outline where this information can be found within the submission (e.g., sections or figure legends), or explain why this information doesn’t apply to your submission:

This information is reported in detail in the Methods section (page 16). Depending on the biological variable of interest, we considered the samples to be the observed single cells when determining the transcriptional profiles (Seruat findMarkers tool). To determine differences in gene expression across the study groups and the placental compartments, we aggregated all of the single-cell data for each cell-type separately (where each individual cell can be considered a technical replicate) and each individual/tissue-location as a biological replicate (DESeq2 analysis). No samples were excluded after all of the data were collected.

Individualized data have been deposited in dbGaP accession number phs001886.v1.p1.

**Statistical reporting**

- Statistical analysis methods should be described and justified
- Raw data should be presented in figures whenever informative to do so (typically when N per group is less than 10)
- For each experiment, you should identify the statistical tests used, exact values of N, definitions of center, methods of multiple test correction, and dispersion and precision measures (e.g., mean, median, SD, SEM, confidence intervals; and, for the major substantive results, a measure of effect size (e.g., Pearson's r, Cohen's d)
- Report exact p-values wherever possible alongside the summary statistics and 95% confidence intervals. These should be reported for all key questions and not only when the p-value is less than 0.05.

Please outline where this information can be found within the submission (e.g., sections or figure legends), or explain why this information doesn’t apply to your submission:

Statistical analyses are described and justified on pages 16 and 17 (Methods, last two subsections). Figure 3B and 3C shows all of the DESeq2 results that passed a multiple test correction; for each gene, we have shown the mean log2 fold change and the 95% confidence interval (CI), as detailed in the caption. Adjusted p-values are described in a color scheme using a colorbar that illustrates the enrichment analyses in Figures S5, S6, S9-S18, as detailed in their legends.

Tables S3, S4 and S5 report all gene expression results and summary statistics for all of the contrasts and for all of the cell-types, including those that are not significant to facilitate comparison and meta-analysis with future studies. Additional p-values and q-values (after adjustments for multiple testing and FDR control) are reported in the captions of Figure 3 and Figure 4 and in the paragraphs (page 7) describing the results in the main body of the manuscript.

Results can also be browsed in the companion website:

<http://placenta.grid.wayne.edu/>.

(For large datasets, or papers with a very large number of statistical tests, you may upload a single table file with tests, Ns, etc., with reference to sections in the manuscript.)

**Group allocation**

- Indicate how samples were allocated into experimental groups (in the case of clinical studies, please specify allocation to treatment method); if randomization was used, please also state if restricted randomization was applied
- Indicate if masking was used during group allocation, data collection and/or data analysis

Please outline where this information can be found within the submission (e.g., sections or figure legends), or explain why this information doesn’t apply to your submission:

- Figure 1A details the sample groups and the study design for the scRNA-seq experiments.

- Figure 4A,D&E illustrate the groups used for follow up analysis using RNA in maternal circulation using previously published data.

- Table S6 details the demographics of the samples.

- Methods section, first paragraph, details inclusion criteria.

- This is not a clinical trial study; therefore, masking and randomization are not applicable.

**Additional data files (“source data”)**

- We encourage you to upload relevant additional data files, such as numerical data that are represented as a graph in a figure, or as a summary table
- Where provided, these should be in the most useful format, and they can be uploaded as “Source data” files linked to a main figure or table
- Include model definition files including the full list of parameters used
- Include code used for data analysis (e.g., R, MatLab)
- Avoid stating that data files are “available upon request”

Please indicate the figures or tables for which source data files have been provided:

The usage of Cellranger, Seurat, and other R packages is detailed in the Methods section.

We also provided the scripts used to execute the pipeline and to run the analyses in this GitHub repository: <https://github.com/piquelab/sclabor>
